# Resection of cervical extra-intraspinal neuromas through the enlarged intervertebral foramen: Results in 34 case series patients

**DOI:** 10.3389/fsurg.2022.945857

**Published:** 2023-04-20

**Authors:** Chuan Chang, Xiao-Ming Che, Ming-Guang Zhang

**Affiliations:** ^1^ Department of Neurosurgery, Huashan Hospital, Shanghai Medical College, Fudan University, Shanghai, China; ^2^ National Center for Neurological Disorders, Shanghai, China; ^3^ Shanghai Key Laboratory of Brain Function and Restoration and Neural Regeneration, Shanghai, China; ^4^ Department of Neurosurgery, Huashan Hospital, Neurosurgical Institute of Fudan University, Shanghai, China; ^5^ Department of Neurosurgery, Huashan Hospital, Shanghai Clinical Medical Center of Neurosurgery, Shanghai, China

**Keywords:** dumbbell neuroma, schwannoma, intervertebral foramen, cervical spine, neurosurgery

## Abstract

**Objective:**

The purpose of this study was to analyze the techniques used to resection cervical extra-intraspinal neuromas (also known as cervical dumbbell neuromas) through the enlarged intervertebral foramen.

**Methods:**

A total of 34 consecutive patients (19 male, 15 female) with cervical dumbbell neuromas reviewed retrospectively between April 2008 and May 2020. Sixteen tumors were found in the intervertebral foramen of C_1_-C_3_, four in C_3_-C_4_, and 14 in C_4_-T_1_. The approach in all cases was to expose the tumors by intermuscular dissection and to remove them through the enlarged intervertebral foramen without excision of any bony structures. However, to expose tumors at different locations, the incisions shall be made accordingly. In this case series, the incisions were made along the posterior border of the sternocleidomastoid muscle for the C_1_-C_3_ tumors and along the anterior border of the muscle for the C_3_-C_4_ tumors. Transverse incisions were required for the C_4_-T_1_ tumors.

**Results:**

Following the mentioned incising approach, all 34 tumors were completely exposed. 31 were completely removed in one stage, and 3 tumors underwent subtotal resection because of brachial plexus nerve adhesion. The vertebral artery and spinal cord were undamaged for all cases. The patients who had total tumor resection showed no sign of recurrence on enhanced magnetic resonance imaging during follow-ups. The status of patients who underwent subtotal resection was stable after radiation therapy. None of the patients developed spinal instability.

**Conclusions:**

Cervical dumbbell neuromas can be exposed and removed through the enlarged intervertebral foramen without causing spinal instability or injury to the spinal cord or vertebral artery. This operative approach can retain the integrity of the structures of spine and should be considered the ideal approach for cervical dumbbell neuromas.

## Introduction

Neuromas are common tumors in the cervical spine or spinal canal, most of which are located in the subdural space. Approximately 15%–18% of cervical neuromas extend into the intervertebral foramen and, often, out of the foramen, forming extra-intraspinal tumors (also known as cervical dumbbell neuromas) (1, 2). Approaches from the posterior midline (3), anterolateral approach (4–6), and posterolateral approach (5–7) are commonly used for the resection of these tumors. The combination of posterolateral and anterolateral approach (7) is occasionally required for the resection of very large tumors. These approaches, however, are very invasive. More importantly, they, in many cases, compromise spinal stability, damage the spinal cord or vertebral artery, and lead to motor and sensory deficit (8).

This study uses the imaging characteristics of the tumors and the local anatomy of necks to resect cervical extra-intraspinal neuromas through the enlarged intervertebral foramen. The clinical results are ideal. Most of cases (31 out of 34) obtained no disadvantages that might occur using traditional operative approaches. This report describes the detailed techniques used in the transforaminal resection of cervical extra-intraspinal neuromas.

## Patients and methods

### Patient data

Our team treated 79 cases of cervical intervertebral foramen involved neuromas from 2008 to 2020. There were 39 intervertebral foramen involved tumors locating the spinal canal, which used posterior midline approach with semi-laminectomy. The 34 cases extra-intraspinal dumbbell neuromas were treated through the enlarged intervertebral foramen. The other 6 patients with huge tumors damaging anterior and posterior column of spine used posterior midline approach with internal fixation.

The study included 34 patients with cervical extra-intraspinal dumbbell neuromas. The preoperative evaluation of each patient comprised magnetic resonance imaging (MRI), computed tomography (CT), cervical spine three-dimensional CT reconstruction, and bilateral CT angiography of the vertebral arteries. In this study, intervertebral foramina tumors whose longest diameters are smaller than 2 centimeters or tumors only extending into spinal canal through intervertebral foramina are excluded from the study. Extra-intraspinal dumbbell neuromas whose longest diameters are longer than 3 centimeters are included in the case series. The preoperative vertebral stability evaluation was conducted based on Spinal Instability Neoplastic Score (SINS standard) for each patient (9) (Table 1).

**Table 1:** The clinical manifestations of the patients.

*P*∼No	Sex/age (year)	Tumor location	Tumor type	Main Symptoms	SINS Score	CervicalJOA score
1	F/46	C_1_-C_2_ R	B	Occipitocervical pain	6	16
2	M/10	C_7_-T_1_ R	B	Numbness and pain in right upper limb	7	15
3	F/16	C_5_-C_6_ L	B	Adynamic and Numbness and pain in left upper limb	6	13
4	M/34	C_1_-C_2_ L	A	Occipitocervical pain and mass in the neck	9	10
5	M/20	C_1_-C_2_ L	A	Occipitocervical pain	7	13
6	M/61	C_1_-C_2_ R	A	Occipitocervical pain	6	15
7	F/44	C_5_-C_6_ L	A	Numbness and adynamic in left upper limb	6	14
8	M/39	C_1_-C_2_ R	A	Occipitocervical pain	6	14
9	M/21	C_3_-C_4_ L	B	Numbness and pain in the neck	6	15
10	M/52	C_3_-C_4_ R	A	Pain and Numbness in the neck	8	12
11	F/44	C_2_-C_3_ L	A	Pain and Numbness in the neck	6	15
12	M/24	C_1_-C_2_ R	A	Occipitocervical pain	7	13
13	F/38	C_3_-C_4_ R	A	Numbness and Pain in the neck	6	14
14	M/64	C_1_-C_2_ R	A	Occipitocervical pain	7	12
15	M/57	C_1_-C_2_ L	A	Occipitocervical pain	6	14
16	M/40	C_4_-C_5_ L	A	Pain and adynamic in the left limb	6	16
17	F/53	C_2_-C_3_ R	A	pain and Numbness in the neck	7	10
18	M/43	C_1_-C_2_ R	C	Occipitocervical pain and mass in the neck	7	13
19	M/43	C_2_-C_3_ L	A	Pain and Numbness in the neck	6	15
20	F/36	C_1_-C_2_ L	C	Occipitocervical pain and mass in the neck	7	15
21	F/47	C_1_-C_2_ R	C	Occipitocervical pain and mass in the neck	7	14
22	F/40	C_1_-C_2_ R	A	Occipitocervical pain	6	16
23	F/41	C_4_-C_5_ L	A	Numbness and adynamic in left upper limb	6	16
24	F/32	C_2_-C_3_ R	C	Numbness and masss in the neck	7	15
25	M/50	C_7_-T_1_ L	B	Numbness and pain in left upper limb	6	15
26	M/37	C_4_-C_5_ R	C	pain and Numbness and adynamic in right upper limb	8	13
27	M/65	C_5_-C_6_ R	C	Numbness and pain and adynamic in right upper limb	8	13
28	F/50	C_3_-C_5_ L	C	Numbness and pain and adynamic in left upper limb/Mass in the neck	13	10
29	M/48	C_4_-C_6_ R	C	Numbness and pain and adynamic in right upper limb	11	14
30	F/51	C_6−_C_7_ R	B	Numbness and pain in right upper limb	7	15
31	F/43	C_1−_C_2_ R	C	Occipitocervical pain and mass in the neck	7	14
32	M/47	C_1−_C_2_ L	A	Occipitocervical pain	6	15
33	M/39	C_5−_C_6_ L	B	Numbness and adynamic in left upper limb	8	14
34	M/63	C_6−_C_7_ R	B	Numbness and pain in right upper limb	7	15

A total of 16 tumors were located in the intervertebral foramina above C_3_, four tumors in C_3_-C_4_, and 14 in C_4_-T_1_. In two patients, the tumor involved two intervertebral foramina (cases 28 and 29). The other tumors involved only one foramen. Among all the patients, the tumors sizes ranged from 3 × 2 × 1.5 cm^3^ to 7 × 7 × 6.5 cm^3^.

Four patients had undergone partial resection of the tumor through a posterior midline approach at other hospitals (cases 1, 3, 26, and 27) before the operation. Two of the three patients with von Recklinghausen's disease (cases 9, 24, and 27) had undergone prior resections of thoracic spinal or subcutaneous tumors. Two patients (cases 20 and 28) had undergone biopsy, one of whom (case 28) had been given local radiotherapy at a local hospital. A transforaminal approach was applied in all patients *via* the enlarged intervertebral foramen. In all patients, the tumors were exposed and removed without laminectomy and spinal fusion.

### Images and classifications

**Figure 1:**
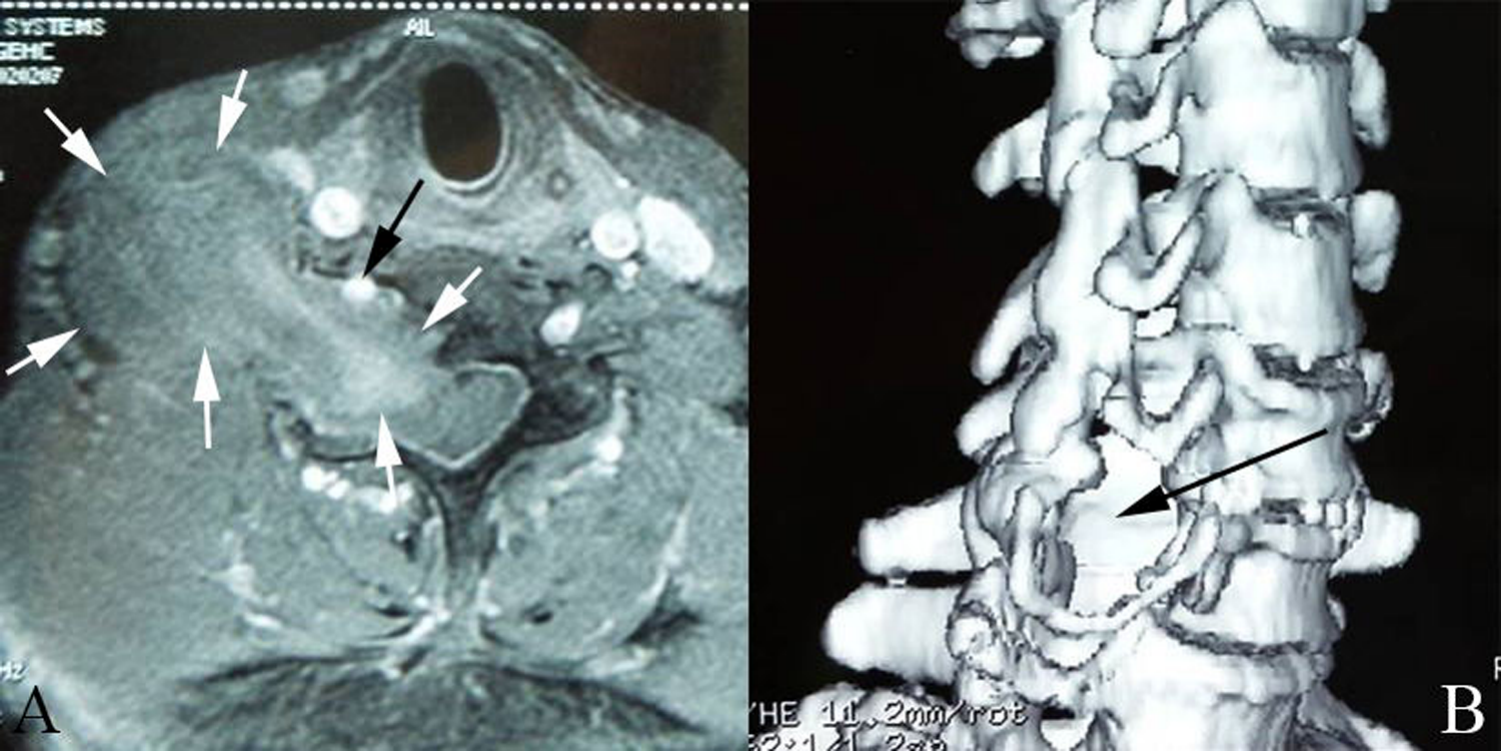
(**A**): the vertebral artery was displacedanteriorly and partially encircled by the tumor. White arrow: tumor; Black arrow:vertebral artery (**B**): Three- dimensional CT reconstruction shows intervertebral foramen was obviously enlarged (black arrow).

On MRIs, the tumor was located in the enlarged vertebral foramen and extending into the spinal canal, eroding on the paravertebral tissue. Due to partial cystic degeneration, Schwannomas enhanced heterogeneously by intravenous contrast. Neurofibromas enhanced more significantly. The vertebral artery was found to be displaced anteriorly and, sometimes, partially encircled by the tumor (see Figure 1A). CT angiography also confirmed the results from MRIs, and furthermore, found that tumors affected the vertebral artery in non-uniform manners and in different extents. Three-dimensional CT reconstruction of the spine demonstrated that the foramen was obviously enlarged, and, at times, caused partial destruction of the vertebral body or lamina plate (see Figure 1B).

**Figure 2:**
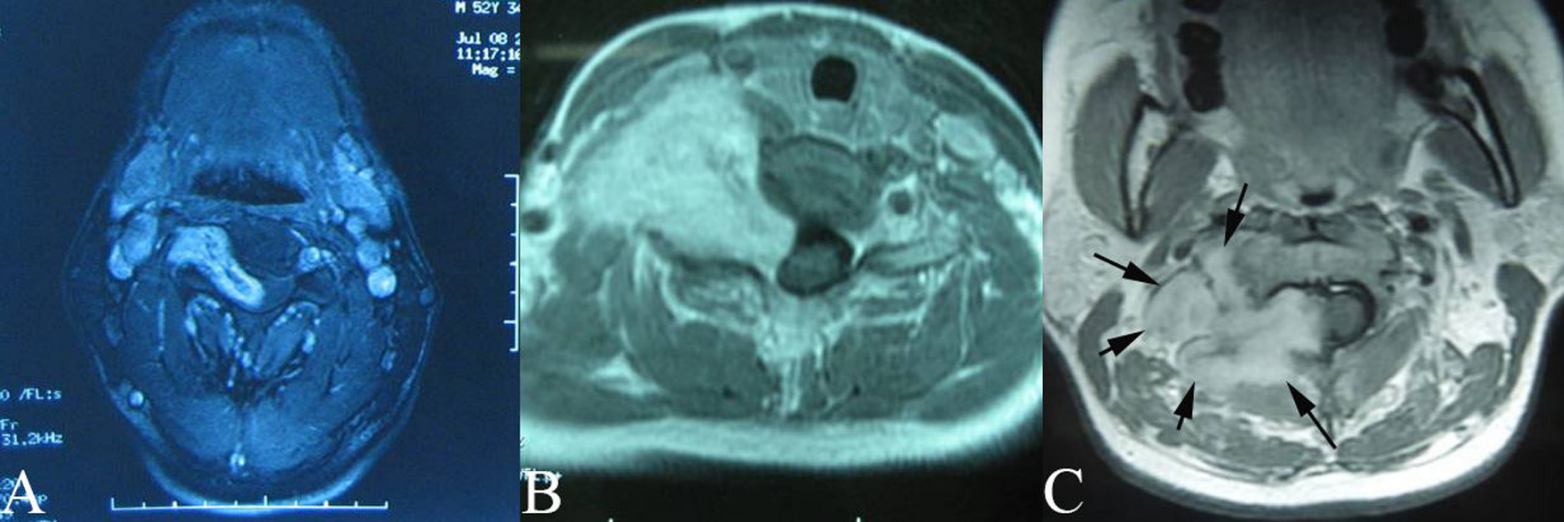
Images and classification of dumbbell cervical neuromas (**A**): type A, intraspinal tumor with extension through the intervertebral foramen to theparaspinal space. (**B**): Type B, Paravertebral tumor with extension to the intervertebralforamen without compressing spinal cord. (**C**): Type C, Giant type which consisted of alarge extra as well as intraspinal tumor compressing spinal cord with destruction of partialvertebral body or lamina plate.

This study referred to the classification of dumb-bell tumors of the spine by Kenneth Eden. The case series tumors had three different types of imaging characteristics: type A, type B, and type C (see Figure 2). Type A is an intraspinal tumor compressing the spinal cord with extension through the enlarged intervertebral foramen to the paravertebral space (see Figure 2A). Type B is a paravertebral tumor with extension to the intervertebral foramen without compression of the spinal cord (see Figure 2B). Type C is a giant type consisting of a large extraspinal and intraspinal tumor with serious destruction of the bony structures and spinal cord compression (see Figure 2C). These imaging characteristics provided the detailed information necessary for preoperative planning. 17 patients were in type A group, 8 in type B group, and 9 in type C group amongst 34 patients in the case series.

### Operative techniques

For the 34 patients with extra-intraspinal dumbbell neuromas, the preoperative vertebral stability evaluation was conducted based on SINS standard for each patient. For type A and B tumors, usually scoring from 7 to 9, internal fixation was unnecessary. For type C tumors, usually scoring from 9 to 13, there were potential risks of vertebral instability. Considering that neuroma did not have osteolytic damage, and the tumors did not have any significant damage to the anterior and posterior column of spine. Enlarged intervertebral foramen approach was applied since it would not damage the bony structure so that vertebral stability may be ensured.

### Position

After the induction of general anesthesia and endotracheal intubation, the patients with C1−C3 or C3−C4 tumors were placed in a lateral position, with the head fixed in a frame and the neck bent 10° to the contralateral side. With this positioning, the incision was in the highest position of the operative field. The patients with C_4_-T_1_ tumors were placed in a supine position with the head rotated 15° to the contralateral side to expose the site of incision.

### Incision and exposure of the tumor

**Figure 3:**
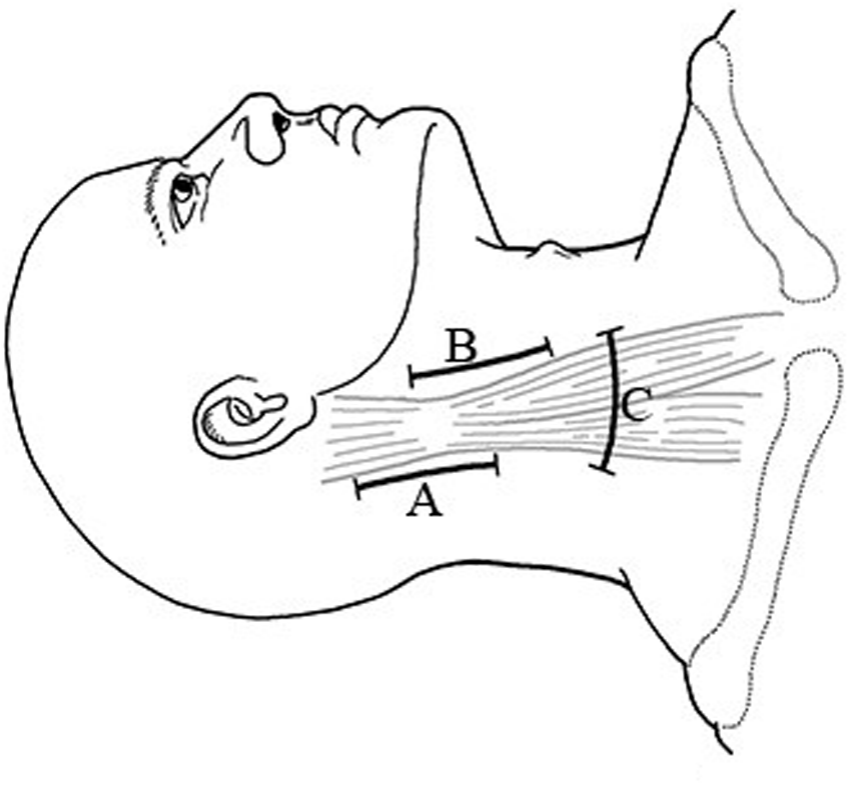
Skin incision (**A**): PMS (**B**): AMS (**C**): Transverse Incision.

For C_1_-C_3_ tumors, incise along the posterior border of the sternocleidomastoid muscle (see Figure 3A). For C_3_-C_4_ tumors, incise along the anterior border of the sternocleidomastoid muscle (see Figure 3B). After retracting sternocleidomastoid muscle, intermuscular dissection was conducted to completely expose the tumors. For C_4_-T_1_ tumors, a transverse incision (see Figure 3C) was made in the appropriate dermatome of the neck based on the location of the tumor, with separation of the muscles and exposure of the tumor *via* intermuscular dissection. Furthermore, for every patient with C_4_-T_1_ tumors, the brachial plexus was carefully identified and protected by consistently monitoring the waves of electrophysiologic monitor to confirm the status throughout the operation.

### Resection of the tumor

**Figure 4:**
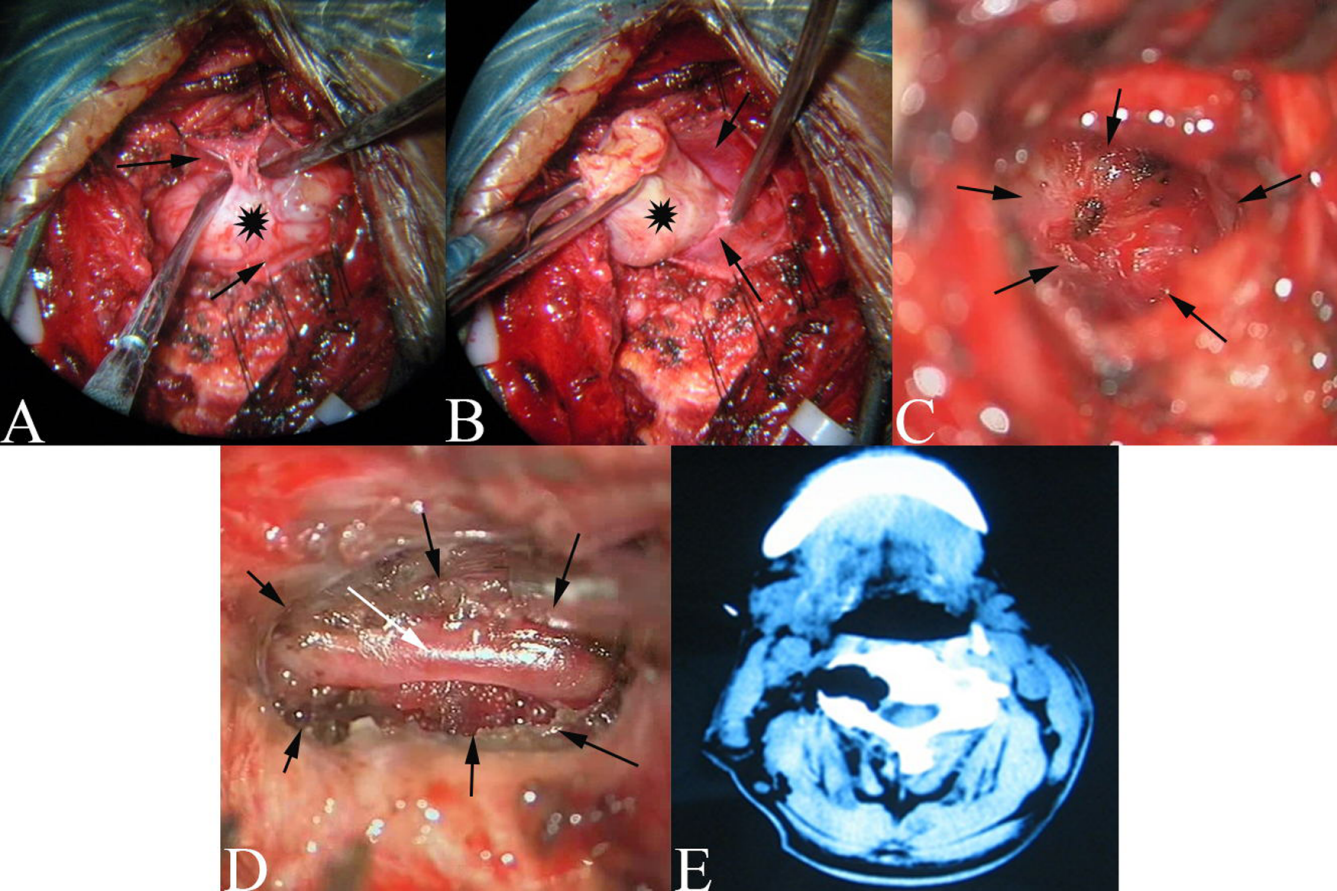
(**A**): the paraspinal tumor was dissected from the capsule, Black Asterisk: tumor; Black Arrow: Capsule of tumor. (**B**): The tumor was dissected in enlarged intervertebral for amen from the capsule. Black Asterisk: tumor; Black Arrow: Capsule of tumor (**C**): Bulging of the translucent membranous. Black arrow: Enlarged interertebral foramen. (**D**): The VA (white arrow)in the enlarged intervertebral foramen (black arrow) after tumor removal. (**E**): The CT of postoperation.

The general process of the operation started with incising longitudinally in the epineurium and the capsule of the tumor. Then, dissect the tumor from the capsule with a peeler (see Figure 4A), and remove it piece by piece. In this process, the paravertebral part of the tumor was removed first. Continue the separation of the tumor within the enlarged intervertebral foramen using similar techniques (see Figure 4B).

Specifically, for type B tumors, the inner end of the tumor was gently pulled out of the foramen while the tumor was being carefully separated from its epineurium or dural nerve cuff. For some of the type A and type C tumors, the intraspinal part of the tumor that compressed the spinal cord was covered by a very thin membranous structure which should be protected throughout the operation to avoid CSF leakage. Remove the intraspinal tumor through the enlarged intervertebral foramen by pulling the tumor gently out of the intraspinal space, and carefully separate it from the very thin capsule. This capsule can be easily dissected while being pulled out of the intraspinal space with the tumor (see Figure 4C). For the other type A and type C tumors, the intraspinal part of the tumor was covered by a thin dura. In some cases, while gently pulling out the tumor, the nerve rootlets might be exposed, or they might be penetrating the tumor. Preserve the nerve rootlets carefully if exposed. Transect the nerve rootlets to remove the tumor completely if penetration happens.

After removing the intraspinal part of tumor, the capsule bulged into the enlarged intervertebral foramen either in the dura or in the translucent membranous structure depending on the size of the tumor. The membranous structure might be thickened arachnoid (see Figure 4C). More importantly, no cerebrospinal fluid (CSF) leaks occurred after removal of these tumors in all the cases.

The parent nerves were often displaced to the side of the tumor. They could be preserved, in most cases, by carefully dissecting the tumor from the capsule of the Schwannoma. Besides, the ganglion of the C_2_ nerve was often tightly adhered to the tumor. In this case, the tumor had to be separated carefully to avoid residues on the ganglion. However, patients with von Recklinghausen's disease, it was difficult to separate the parent nerve from the neurofibroma. Thus, the parent nerves had to be sacrificed to guarantee complete tumor resection. For the one neurofibroma originating from the brachial plexus nerve root, the tumor had to be subtotal resected to preserve brachial nerve function (case 27).

The parent nerves between C_4_-T_1_ are main components of the brachial plexus nerve. Therefore, to protect parent nerves, the incision of the capsule of the tumor should be made parallel. Separate the tumor carefully along the interface with the parent nerve. This process was under consistent electrophysiologic monitoring.

While separating the tumor from its capsule, a micro-current bipolar coagulator was used to coagulate bleeding to minimize injury to the parent nerve. After completely resecting the dumbbell tumor, the capsule was sutured to prevent CSF leakage.

### Protection of the vertebral artery

The vertebral artery was usually displaced anteriorly without direct invasion by the tumor. By separating the tumor carefully from the thin capsule, injury to the vertebral artery was prevented. For type C tumors and neurofibroma from von Recklinghausen's disease, the vertebral artery was often surrounded circumferentially by the tumor with thin epineurium or capsule. Therefore, during the dissection of the tumor from the capsule *via* the enlarged vertebral foramen, the position and direction of the vertebral artery was located using an ultrasonic Doppler probe to give more detailed information for further protection. After completing the removal of the tumor, the vertebral artery could be seen within the enlarged intervertebral foramen in three patients (see Figure 4D; cases 21, 24, and 28).

### Protection of bony vertebral structures

All tumors were resected *via* the enlarged intervertebral foramen exposure without a laminectomy or the destruction of any bony structures. It was crucial to select the optimal position of the incision to expose the tumor in the enlarged intervertebral foramen and its intraspinal component, allowing the tumor to be resected without the incision or destruction of any bony structures of the spine.

Postoperative patients of Type A and B used cervical brace for at least one month, the patient with Type C used cervical brace more than 3 months.

## Results

This case series consisted of 15 female and 19 male patients between the ages of 10 and 65 (average 42.3 years). The preoperative duration of symptoms ranged from 3 weeks to 120 months (average 24 months). The clinical manifestations had three primary types of symptoms including local symptoms involving pain in the neck or shoulder and/or a neck mass, radicular symptoms with either pain or numbness in the arms in the neural distribution of the involved nerves, and medullary symptoms with ipsilateral or bilateral limb weakness (10). Three patients who experienced a decrease in ipsilateral upper limb strength after incomplete resection of the tumor in other hospitals were also included (see Table 1, cases 3, 26, and 27).

All tumors were exposed completely, with 31 being completely removed in one stage and 3 tumors undergoing subtotal resection because of brachial plexus nerve adhesion. Of the 30 Schwannoma resected, 25 parents' nerves were preserved. However, of the three neurofibroma resected, preservation of parent nerve was successful in one case (case 27). The bony structures of the involved intervertebral foramina were preserved, and cervical internal fixation or bone grafting was not necessary. The vertebral arteries were preserved carefully in all patients. There was no postoperative CSF leakage. A total of 30 patients reported improvement or stabilization of their symptoms. Two patients reported transient ipsilateral ear skin pain (cases 20 and 22) which resolved within two months, and two others experienced temporary decreased strength in their ipsilateral upper limb (class IV to class III) (cases 3 and 27). Pathological findings included Schwannoma in 30 patients, neurofibromas in three, and a ganglion neurofibroma in one.

**Figure 5:**
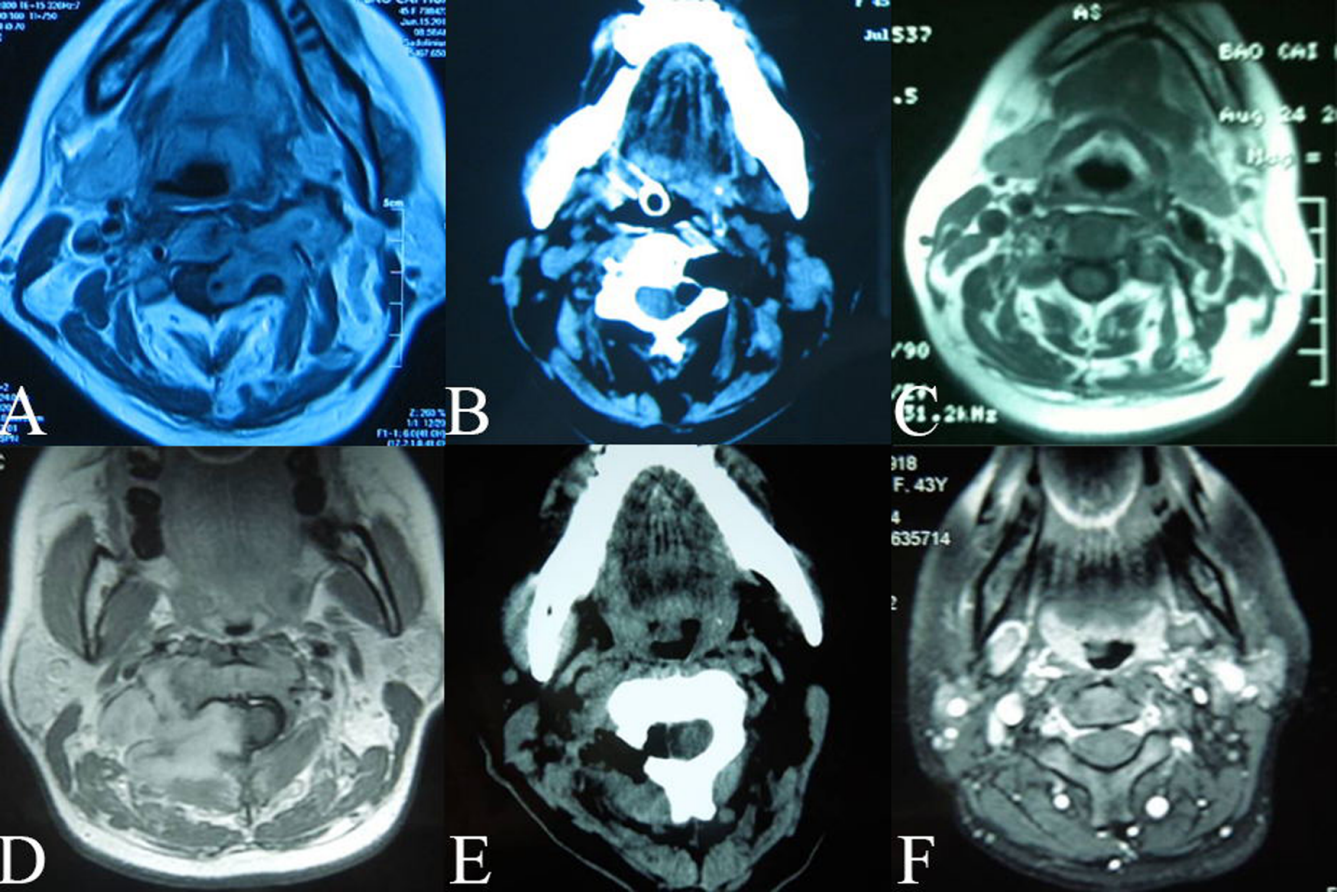
Preoperative and postoperative follow-up images (**A. D**): Preoperation images (**B. E**): Postoperative CTs: totall removal of tumors (**C. F**): Postoperative follow-up MRIs: no tumor residual or recurrent.

**Figure 6:**
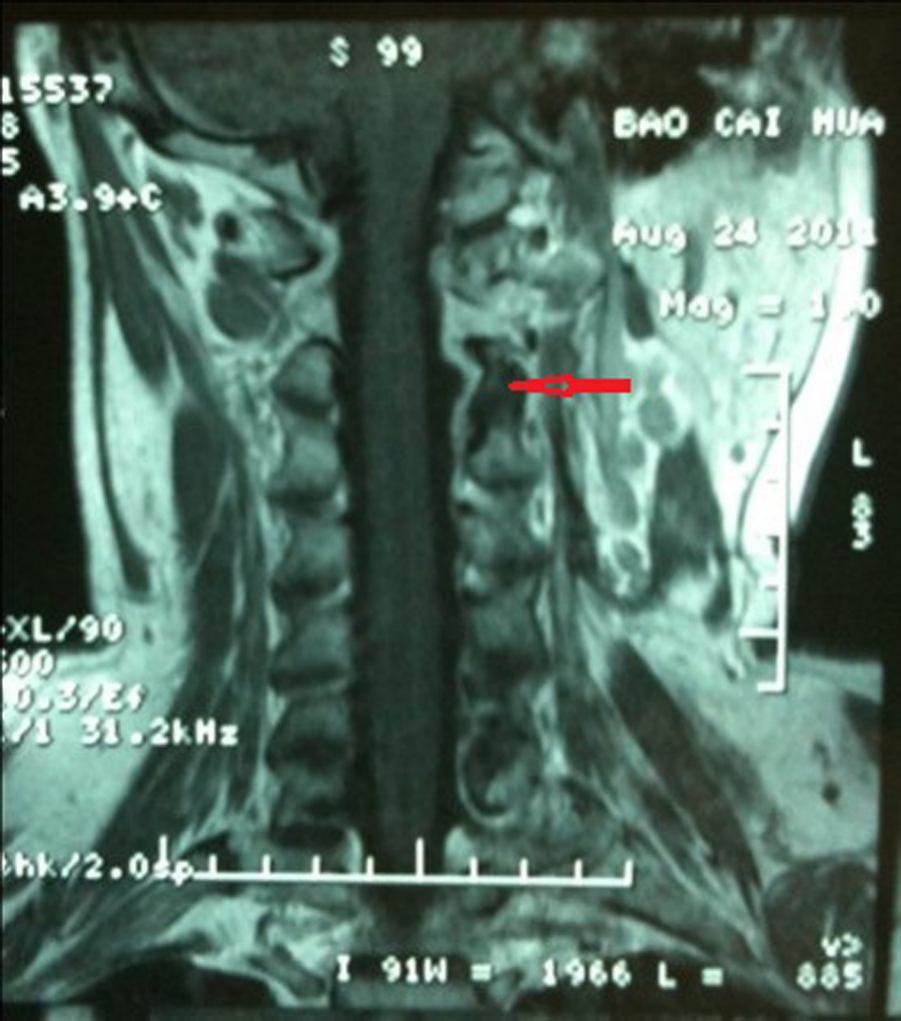
MRIs of the postoperative cervical spine.

The follow-up duration was 15–159 months. During follow-up, enhanced MRI showed no recurrence in patients who underwent complete tumor resection (see Figure 5). The tumor residues of the patients who underwent subtotal resection had not enlarged six months after the operation, but there was no further follow-up for this patient after that time. MRIs and three-dimensional CT reconstruction of the cervical spine showed preservation of the structural integrity of all cervical joints (See Figure 6), and none of the patients complained about the symptoms of cervical spinal instability. One patient with a decrease in upper limb strength (class IV) returned to their preoperative level, while the other showed only mild improvement. All patients were able to carry out normal neck activities. The cervical Japanese Orthopedic Association scores of all patients are shown in Table 2.

**Table 2:** The results of the patients.

*P*∼No	Sex/age (year)	Tumor location	Tumor type	Skin Incision Outcome	Pathology	CervicalJOA scorePre/post Op
1	F/46	C_1_-C_2_ R	B	Longitudinal (PMS) Total Resection	Schwannoma	16/17
2	M/10	C_7_-T_1_ R	B	Transverse Total Resection	Schwannoma	15/17
3	F/16	C_5_-C_6_ L	B	Transverse Total Resection	Schwannoma	13/16
4	M/34	C_1_-C_2_ L	A	Longitudinal (PMS) Total Resection	Schwannoma	10/16
5	M/20	C_1_-C_2_ L	A	Longitudinal (PMS) Total Resection	Schwannoma	13/17
6	M/61	C_1_-C_2_ R	A	Longitudinal (PMS) Total Resection	Schwannoma	15/17
7	F/44	C_5_-C_6_ L	A	Transverse Total Resection	Schwannoma	14/16
8	M/39	C_1_-C_2_ R	A	Longitudinal (PBS) Total Resection	Ganglionneurofibroma	14/17
9	M/21	C_3_-C_4_ L	B	Longitudinal (ABS) Total Resection	Neurofibroma	15/17
10	M/52	C_3_-C_4_ R	A	Longitudinal (ABS) Total Resection	Schwannoma	12/16
11	F/44	C_2_-C_3_ L	A	Longitudinal (PBS) Total Resection	Schwannoma	15/17
12	M/24	C_1_-C_2_ R	A	Longitudinal (PBS) Total Resection	Schwannoma	13/17
13	F/38	C_3_-C_4_ R	A	Longitudinal (ABS) Total Resection	Schwannoma	14/17
14	M/64	C_1_-C_2_ R	A	Longitudinal (PBS) Total Resection	Schwannoma	12/16
15	M/57	C_1_-C_2_ L	A	Longitudinal (PBS) Total Resection	Schwannoma	14/17
16	M/40	C_4_-C_5_ L	A	Transverse Subtotal Resection	Schwannoma	16/17
17	F/53	C_2_-C_3_ R	A	Longitudinal (PBS) Total Resection	Schwannoma	10/15
18	M/43	C_1_-C_2_ R	C	Longitudinal (PBS) Total Resection	Schwannoma	13/16
19	M/43	C_2_-C_3_ L	A	Longitudinal (PBS) Total Resection	Schwannoma	15/17
20	F/36	C_1_-C_2_ L	C	Longitudinal (PBS) Total Resection	Schwannoma	15/16
21	F/47	C_1_-C_2_ R	C	Longitudinal (PBS) Total Resection	Schwannoma	14/16
22	F/40	C_1_-C_2_ R	A	Longitudinal (PBS) Total Resection	Schwannoma	16/17
23	F/41	C_4_-C_5_ L	A	Transverse Subtotal Resection	Schwannoma	16/17
24	F/32	C_2_-C_3_ R	C	Longitudinal (PBS) Total Resection	Neurofibroma	15/17
25	M/50	C_7_-T_1_ L	B	Transverse Total Resection	Schwannoma	15/17
26	M/37	C_4_-C_5_ R	C	Transverse Total Resection	Schwannoma	13/16
27	M/65	C_5_-C_6_ R	C	Transverse Subtotal Resection	Neurofibroma	13/14
28	F/50	C_3_-C_5_ L	C	Transverse Total Resection	Schwannoma	Oct-16
29	M/48	C_4_-C_6_ R	C	Transverse Total Resection	Schwannoma	14/17
30	F/51	C_6−_C_7_ R	B	Transverse Total Resection	Schwannoma	15/17
31	F/43	C_1−_C_2_ R	C	Longitudinal (PBS) Total Resection	Schwannoma	14/17
32	M/47	C_1−_C_2_ L	A	Longitudinal (PBS) Total Resection	Schwannoma	15/17
33	M/39	C_5−_C_6_ L	B	Transverse Total Resection	Schwannoma	14/17
34	M/63	C_6−_C_7_ R	B	Transverse Total Resection	Schwannoma	15/17

## Discussion

**Figure 7:**
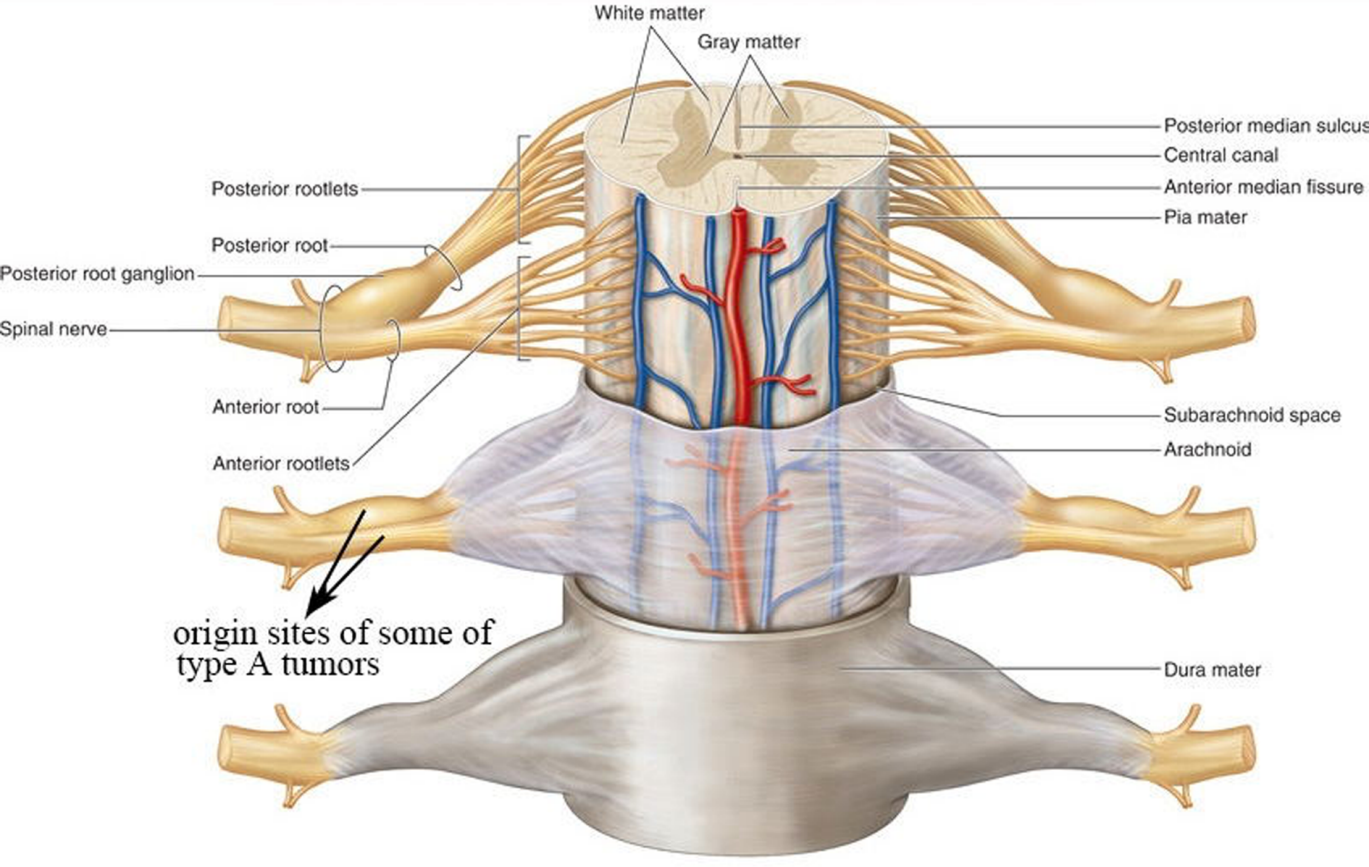
Origin sites of some of type A tumors.

Relevant literature has identified two origins of dumbbell neuromas in the cervical spinal region. Subdural tumors extend through the dura sleeve of the nerve root and into the intervertebral foramen to form a dumbbell-shaped tumor that spans the dura space (1, 11). However, previous reports do not elaborate the exact origin of the subdural tumor and its relationship with the arachnoid. In the study, the inner end of some dumbbell neuromas were covered by a thickened arachnoid (see Figure 4C). Therefore, it suggested that some of the tumors originated from the posterior root ganglion or the anterior root out of the arachnoid in the dura sleeve of the nerve root (see Figure 7). The second proposed origin of dumbbell neuromas is the paravertebral segments of the spinal nerves (3) that are located entirely in the epidural space, with the tumors extending into the intervertebral foramen (2, 5,6, 12–15). In the study, 8 tumors (see Figure 2B) were as the reported in the second proposal.

Several classifications of dumbbell neuroma guide surgeons in choosing the most appropriate surgical approach (7, 12, 14), thereby, improving the effect of surgical treatment with less injury. All types of tumors under the Eden classification, some types of tumor within the Asazuma classification (type IIb, IIc, IIIb, and VI), and type A tumors can be resected using the enlarged intervertebral foramen approach.

Based on tumor imaging and intraoperative findings, the study divided the tumors in the sample group into three types: type A, B, and C, to guide surgical treatment. Type A tumors compress the spinal cord directly and extend into the intervertebral foramen, and resection of these tumors should focus on preventing CSF leakage and injury to the spinal cord. Type B tumors are larger and are located paravertebrally, extending into the intervertebral foramen. During the resection, the nerves and vertebral artery around these tumors should be carefully protected. For type C tumors, it is important to protect both the vertebral artery, which is often encased by the tumor, and to avoid injury of the spinal cord.

The operative approaches most commonly used for resecting cervical dumbbell neuromas are the posterior midline approach (3) and the anterolateral approach (4–7, 13). These approaches allow the removal most of a cervical dumbbell neuroma in one stage. However, the posterior midline approach may also cut open the involved intervertebral joints with hemilaminectomy or laminectomy, and the posterolateral approach for the resection of paraspinal tumors requires exposure of the levator scapulae muscles and the middle and posterior scalene muscles from the posterior tubercle of the adjacent transverse process (5–7). The common disadvantage of these approaches is that they are very invasive and result in more bleeding. In addition, they may lead to instability of the spine, injury to the vertebral artery, and restricted cervical mobility caused by cervical fixation. For large tumors, the anterolateral approach may need to be combined with the posterior midline approach, or the operation may need to be staged. The advantage of these approaches is that they may expose and protect the brachial plexus and vertebral artery. However, the anterolateral approach is very invasive and often results in bleeding from the venous plexus or vertebral artery, and it may damage the sympathetic chain and accessory nerve.

The study used cross-sectional MRI images to easily identify dumbbell neuromas located in the enlarged intervertebral foramen and extending into the paraspinal and spinal canal. Paraspinal tumors were covered by regional muscles and bulging skin. The choice of incision was made based on the location of the tumor: a vertical incision along the posterior border of the sternocleidomastoid muscle was used to expose tumors located in the enlarged intervertebral foramen of C_1_-C_3_, and a vertical incision along the anterior border of the sternocleidomastoid muscle was used in C_3_-C_4_ tumors. For C_4_-T_1_ tumors, a transverse incision in the neck was optimal. Paraspinal tumors can be exposed by intermuscular dissection. In the process of exposing the tumor, it is vital that the nerves, especially the brachial plexus, are identified and protected. Following such an approach to expose paraspinal tumors is less invasive, and causes less dissection of the soft tissues and other structures of the neck. However, protecting the brachial plexus may be challenging during the resection of large C_4_-T_1_ tumors because of narrow exposures, so that electrophysiological monitoring is necessary to signal risks before damaging the nerves.

Using this transforaminal approach, tumors were easily removed through the enlarged intervertebral foramen. Although the intraspinal tumor compressed the spinal cord, it was attainable to safely and completely separate the tumor with enough care in the whole process. The approach could easily identify the structural characteristics of the parent nerves and tumors, as that the parent nerve bundles are often located on the surface of the schwannomas (16) which could be hard to identify using other approaches. Neurofibromas from von Recklinghausen's disease were easily removed through the enlarged intervertebral foramen exposure, but it was challenging to preserve the parent nerves. For tumors associated with the brachial plexus, subtotal resection of the tumor should be performed to preserve the function of the brachial plexus.

The stability of the cervical spine is always a concern after the resection of cervical dumbbell neuromas using the posterolateral approach. Fusion or plate fixation of the cervical spine has become common to prevent long-term instability. However, fixation leads to common problems with postoperative cervical mobility and increase the costs of the operation. In the study, resection of the cervical dumbbell neuromas through the enlarged intervertebral foramen was carried out completely without damaging the surrounding bony and joint structures. This approach maintained the structural integrity of the cervical spine and preserved postoperative cervical mobility. Patients treated using this approach showed no sign of developing spinal instability. Patients in the follow-up had normal cervical mobility, with postoperative three-dimensional CT reconstruction and cross-sectional imaging showing no deformities of the spinal structures.

The vertebral artery is often displaced anteriorly or encased by a large cervical extra-intraspinal neuroma. When using the anterolateral and posterolateral approach to treat the dumbbell neuromas, the vertebral artery is dissected before resecting the tumor (5). However, these approaches are likely to injure the vertebral artery, potentially increasing the morbidity of the operation (4, 5). In the study, the tumors were dissected carefully *via* exposure through the enlarged intervertebral foramen, thereby avoiding the dissection of the vertebral artery. Preoperative cross-sectional imaging is also necessary to identify the adhesion of the tumor to the vertebral artery. The imaging is especially important for large type C tumors in terms of the vertebral artery. Using preoperative MRI to identify the anatomic position relation and Doppler probe during the operation to scrutinize the position of the vertebral artery, these types of neuroma can be successfully resected through the enlarged intervertebral foramen without injury.

## Conclusion

Removing tumors through enlarged intervertebral foramen, cervical dumbbell neuromas were fully exposed and removed completely resected, with no excision of the surrounding bony structures or transection of the muscles, thereby maintaining spinal stability. Through careful dissection of the tumor, the vertebral artery and spinal cord were protected from injury. This operative approach for the resection of cervical dumbbell tumors should be considered with priority for the larger cervical dumbbell neuromas.

## Data Availability

The original contributions presented in the study are included in the article/Supplementary Material, further inquiries can be directed to the corresponding author/s.
